# Prevalence and Factors Associated With Urogenital Bacterial Colonization Among Women With Preterm Labor at a Tertiary Hospital in Southwestern Uganda: A Cross-Sectional Study

**DOI:** 10.7759/cureus.112011

**Published:** 2026-07-03

**Authors:** Wilson Birungi, Stuart Turanzomwe, Onesmus Byamukama, Bawakanya M Stephen, Taseera Kabanda, Musa Kayondo, Rogers Kajabwangu

**Affiliations:** 1 Obstetrics and Gynecology, Mbarara University of Science and Technology, Mbarara, UGA; 2 Obstetrics and Gynecology, Kabale University, Kabale, UGA; 3 Obstetrics and Gynecology, Mbarara Regional Referral Hospital, Mbarara, UGA; 4 Microbiology and Parasitology, Mbarara University of Science and Technology, Mbarara, UGA

**Keywords:** genital tract, pregnancy, preterm labour, southwestern uganda, urinary tract, urogenital bacterial colonization

## Abstract

Introduction: Urogenital bacterial colonization is common during pregnancy and is associated with preterm labor and preterm births. Physiological, hormonal, and immunological changes of pregnancy increase the susceptibility to colonization of the urogenital tract by pathogenic bacteria, which may contribute to adverse pregnancy outcomes. This study aimed to estimate the prevalence of urogenital bacterial colonization and identify factors associated with it among women with preterm labor at Mbarara Regional Referral Hospital. Materials and methods: This was a cross-sectional study involving 156 women with preterm labor who were consecutively enrolled between November 2022 and April 2023. Data on independent variables were collected using an interviewer-guided structured questionnaire. Urine samples and endocervical and high vaginal swabs were collected and processed for microbiological culture to detect bacterial colonization. We used modified Poisson regression to identify factors associated with bacterial colonization. Results: Out of the 156 women with preterm labor enrolled in this study, 85 (54.5%) had urogenital bacterial colonization. The mean age of the participants was 25.5 ± 5.77 years. Most of the participants were referred from other health facilities (n = 120, 76.9%) and had a history of abnormal vaginal discharge (n = 117, 75%). Factors independently associated with bacterial colonization included age <20 years (aPR 1.66, 95% CI: 1.23-2.25, p = 0.001) and abnormal vaginal discharge (aPR 1.79, 95% CI: 1.12-2.87, p = 0.016). Conclusions: Urogenital bacterial colonization was common among women with preterm labor in southwestern Uganda. Younger maternal age (<20 years) and abnormal vaginal discharge were independently associated with urogenital bacterial colonization. These findings highlight the need for increased clinical suspicion and targeted evaluation of urogenital bacterial colonization, including microbiological culture and antibiotic susceptibility testing of urinary tract and genital tract specimens, particularly among pregnant women with preterm labor, who are at higher risk.

## Introduction

Preterm birth, defined as delivery before 37 completed weeks of gestation, remains a major global public health problem [1]. Approximately 15 million babies are born preterm each year, and nearly 1 million die from complications related to prematurity [1,2]. In addition to neonatal mortality, preterm birth is associated with significant short- and long-term morbidity, including respiratory, neurological, and developmental complications.

Urogenital bacterial colonization is common during pregnancy and is estimated to contribute up to one third of all preterm births [3]. Physiological, hormonal, and immunological changes that occur during pregnancy increase susceptibility to colonization of the urogenital tract by potentially pathogenic microorganisms. These changes are associated with altered immune responses, including reduced activity of some inflammatory mediators such as interleukin-2, which may facilitate microbial growth and persistence within the urogenital tract [4,5]. Although colonization may be asymptomatic, it can progress to infection and trigger inflammatory pathways implicated in the initiation of preterm labor. Microbial invasion stimulates the release of pro-inflammatory cytokines, including interleukin-1β and tumor necrosis factor, leading to increased prostaglandin production, activation of matrix metalloproteinases, cervical ripening, membrane weakening, and uterine contractions, culminating in preterm labor [5,6].

The prevalence of urogenital bacterial colonization among women with preterm labor varies across settings. Studies have reported prevalences ranging from 36.5% to 67.8% in India, 65% in the United States, 31% in Ethiopia, and 26.7% in Kenya [7-10]. Colonization of the urogenital tract has been associated with adverse maternal and neonatal outcomes, including preterm birth, chorioamnionitis, fetal distress, intrauterine fetal demise, postpartum endometritis, and wound sepsis after cesarean section or episiotomy [11,12].

Several factors have been associated with urogenital bacterial colonization during pregnancy. These include premature rupture of membranes, bacterial vaginosis, vulvovaginal candidiasis, viral infections, multiple sexual partners, and low socioeconomic status [5,13]. However, the factors associated with urogenital bacterial colonization among women presenting with preterm labor may vary across populations because of differences in sociodemographic characteristics, sexual behaviors, healthcare access, and microbial ecology.

In Uganda, data on factors associated with urogenital bacterial colonization among women with preterm labor are limited. Understanding these factors is important for identifying high-risk women and informing targeted screening, prevention, and treatment strategies. Therefore, this study aimed to estimate the prevalence of urogenital bacterial colonization and identify factors associated with it among women with preterm labor at Mbarara Regional Referral Hospital (MRRH) in southwestern Uganda.

This article was previously presented as a conference abstract at the 20th Annual Scientific Conference of the Association of Obstetricians and Gynecologists of Uganda on November 14, 2025.

## Materials and methods

Study design, setting, and population

This was a hospital-based cross-sectional study conducted among women with preterm labor admitted to the maternity ward at MRRH between November 1, 2022, and April 30, 2023. MRRH is a tertiary hospital owned and funded by the government of Uganda through the Ministry of Health. It cares for about 12,000 pregnant women annually, including those with preterm labor [14,15]. Our study population comprised women admitted with preterm labor to the maternity ward of MRRH. We included all women with a diagnosis of preterm labor, as evidenced by the presence of two or more palpable uterine contractions in 10 minutes and cervical dilatation ≥2 cm at a gestational age of 26 to 36 weeks and six days, which was calculated from the first day of the last normal menstrual period or first trimester ultrasound scan.

Ethical approval was obtained from the Mbarara University of Science and Technology Research Ethics Committee (MUST-2022-563), and the study was registered with the Uganda National Council for Science and Technology (HS3075ES).

Sample size and sampling

The sample size was calculated using the OpenEpi online software (OpenEpi Project, GA, USA) to estimate a single population proportion. A finite population correction was applied based on an estimated population of 240 women with preterm labor expected to present to MRRH over a six-month period. The expected frequency of bacterial colonization was set at 67.8%, based on a study conducted among women with preterm labor in India [7]. After accounting for a 10% non-response rate, the final sample size was 156 participants. Eligible women were enrolled consecutively until the required sample size was achieved.

Data collection and study variables

Data were collected using a pretested, interviewer-guided structured questionnaire developed after a review of the relevant literature (Appendix 1). The questionnaire was reviewed by the study supervisors and experts in obstetrics and gynecology to assess its content validity. It was subsequently pretested among 10 women with preterm labor to evaluate its clarity, completeness, and appropriateness. Based on the findings of the pretest, minor revisions were made to improve wording and completeness of the questionnaire before commencement of the main study. The women who participated in the pretest were not included in the final analysis. Information obtained included sociodemographic characteristics, obstetric history, and medical factors that may be associated with urogenital bacterial colonization. The dependent variable was urogenital bacterial colonization. This was defined as the isolation of pathogenic bacteria from urine, high vaginal swab, or endocervical swab specimens. Urinary bacterial colonization was defined as growth of a pathogenic organism at a concentration of ≥105 CFU/mL of midstream urine. In contrast, genital tract bacterial colonization was defined as the isolation of at least one pathogenic bacterial colony from a high vaginal or endocervical swab. Participants with either urinary or genital tract bacterial colonization were classified as having urogenital bacterial colonization.

Independent variables included sociodemographic factors (age, parity, residence, education level, and number of sexual partners), obstetric factors (gestational age, number of antenatal care visits, preterm premature rupture of membranes, and number of vaginal examinations), and medical factors (HIV status, diabetes mellitus, antibiotic use, and abnormal vaginal discharge). Information was obtained through participant interviews and verified from antenatal records. Testing was performed according to the Ministry of Health guidelines for participants without a documented HIV test result within the preceding three months.

After obtaining informed consent, participants were interviewed in a private clinical room. Clean-catch midstream urine samples, high vaginal swabs, and endocervical swabs were collected aseptically by the principal investigator or trained research assistants. Samples were transported to the laboratory within 30 minutes of collection for microbiological culture and bacterial identification.

Specimens were inoculated onto 5% sheep blood agar, MacConkey agar, mannitol salt agar, and modified Thayer-Martin agar to isolate aerobic bacteria. The inoculated media were incubated aerobically at 37°C for 24-72 hours. Modified Thayer-Martin agar plates were incubated in a humidified atmosphere containing 5% carbon dioxide.

Bacterial isolates were identified using conventional phenotypic methods, including colony morphology, Gram staining, and standard biochemical tests. *Staphylococcus aureus* was identified by positive catalase, coagulase, and DNase tests, while Gram-negative bacilli were identified and differentiated using urease, citrate utilization, oxidase, and triple sugar iron tests. Gram staining was performed according to standard microbiological procedures, and stained smears were examined microscopically under oil immersion (×100) to determine the Gram reaction and bacterial morphology.

Quality assurance was maintained through both internal and external quality control procedures. The MRRH microbiology laboratory participates in external quality assurance programs coordinated by the Uganda National Health Laboratory Services and the American College of Pathologists. Internal quality control was performed using standard reference strains, including *Staphylococcus aureus* ATCC 25923 for Gram-positive bacteria and *Escherichia coli* ATCC 25922 for Gram-negative bacteria. Additionally, every tenth specimen was re-examined at the EpiCentre Laboratory, a Level III accredited reference laboratory in Mbarara, for external quality control.

Participants with positive culture results were contacted and informed of their laboratory findings. The laboratory results were also communicated to the attending clinical team to facilitate appropriate management in accordance with MRRH's standard treatment protocols.

Data management and analysis

Completed questionnaires were checked daily for completeness and consistency before data entry into RedCap® software (Vanderbilt University, Nashville, TN, USA). The data were securely stored, electronically backed up, and exported to Stata® version 17 (StataCorp. Stata Statistical Software: Release 17. College Station, TX: StataCorp LLC; 2021) [16] for cleaning and analysis. Participant characteristics were summarized using means and standard deviations for continuous variables and frequencies with percentages for categorical variables.

The prevalence of urogenital bacterial colonization was calculated as the proportion of women with a positive bacterial culture from urine, endocervical swab, and/or high vaginal swab specimens, irrespective of whether colonization was detected in the urinary tract, genital tract, or both, and was reported with 95% CIs. Participant characteristics were summarized using means and standard deviations for continuous variables and frequencies with percentages for categorical variables. In bivariate analysis, differences in continuous variables were assessed using the independent-samples Student’s t-test. In contrast, associations between categorical variables and urogenital bacterial colonization were assessed using Pearson’s chi-square test or Fisher’s exact test as appropriate. Variables with a p-value <0.20 at bivariate analysis and those considered biologically plausible were included in the multivariable modified Poisson regression model with robust standard errors to estimate adjusted prevalence ratios (aPRs) and their corresponding 95% CIs. Statistical significance was considered at a p-value <0.05.

## Results

A total of 156 women with preterm labor were enrolled in the study. The mean age of participants was 25.5 ± 5.8 years. Most of the participants were aged between 20 and 34 years (n = 126, 80.8%), had a parity of fewer than four deliveries (n = 137, 87.8%), and resided in rural areas (n = 91, 58.3%). There were no statistically significant differences in age, parity, residence, educational attainment, marital status, number of sexual partners, or referral status between women with and without urogenital bacterial colonization (Table 1).

**Table 1:** Baseline sociodemographic characteristics of women with preterm labor by urogenital bacterial colonization status SD: standard deviation, χ2: Pearson's chi-square test, t: independent samples Student’s t-test, referral status: women referred from other health facilities

Variable	Total N = 156	Colonization N = 85	No colonization N = 71	Test statistic	p-value
	n/N (%)	n/N (%)	n/N (%)		
Age (mean ± SD)	25.5 ± 5.77	25.31 ± 5.57	25.73 ± 6.04	t = 0.46	0.648
Age category (years)				χ^2^ = 3.74	0.154
<20	17 (10.9)	13 (15.3)	4 (5.6)		
20-34	126(80.8)	65 (76.5)	61 (85.9)		
≥35 years	13 (8.3)	7 (8.2)	6 (8.5)		
Parity				χ^2^ = 0.10	0.750
<4	137 (87.8)	74 (87.1)	63 (88.7)		
≥4	19 (12.2)	11 (12.9)	8 (11.3)		
Residence				χ^2^ = 1.37	0.243
Urban area	65 (41.7)	39 (45.9)	26 (36.6)		
Rural area	91 (58.3)	46 (54.1)	45 (63.4)		
Highest level of education				χ^2^ = 0.65	0.737
Primary	77 (49.4)	42 (49.4)	35 (49.3)		
Secondary	54 (34.6)	31 (36.5)	23 (32.4)		
Tertiary/university	25 (16.0)	12 (14.1)	13 (18.3)		
Marital status (N = 155)				χ^2^ = 1.42	0.641
Single	20 (12.9)	10 (11.8)	10 (14.3)		
Married/living with a partner	135 (87.1)	75 (88.2)	60 (85.7)		
Multiple sexual partners				Fisher’s exact test	0.322
No	148 (94.9)	82 (96.5)	66 (93.0)		
Yes	8 (5.1)	3 (3.5)	5 (7.0)		
Referral status				χ^2^ = 0.38	0.538
No	36 (23.1)	18 (21.2)	18 (25.4)		
Yes	120 (76.9)	67 (78.8)	53 (74.6)		

Medical and obstetric characteristics of participants

Most participants were HIV negative (92.3%, n = 144); had no history of diabetes mellitus (n = 153, 98.1%); had not used antibiotics in the preceding seven days (n = 128, 82.1%); reported abnormal vaginal discharge (n = 117, 75.0%); and had undergone two or fewer vaginal examinations at enrollment (n = 115, 73.7%). Nearly half of the participants had premature rupture of membranes (n = 73, 46.8%), while 50.0% (n = 78) had attended at least four antenatal care visits. Women with urogenital bacterial colonization were more likely to report abnormal vaginal discharge than those without colonization (83.5% (n = 71) vs. 64.8% (n = 46), p = 0.007) (Table 2).

**Table 2:** Medical and obstetric characteristics by urogenital bacterial colonization status ANC: antenatal care, HIV: human immunodeficiency virus, χ^2^: Pearson's chi-square test

Variable	Total N = 156	Colonization N = 85	No colonization N = 71	Test statistic	p-value
	n/N (%)	n/N (%)	n/N (%)		
HIV status				χ^2^ = 0.11	0.745
Negative	144 (92.3)	79 (92.9)	65 (91.6)		
Positive	12 (7.7)	6 (7.1)	6 (8.4)		
Diabetes mellitus				Fisher’s exact test	0.669
No	153 (98.1)	83 (97.6)	70 (98.6)		
Yes	3 (1.9)	2 (2.4)	1 (1.4)		
Antibiotic in the past 7 days				χ^2^ = 0.28	0.599
No	128 (82.1)	71 (83.5)	57 (80.3)		
Yes	28 (17.9)	14 (16.5)	14 (19.7)		
Abnormal vaginal discharge				χ^2^ = 7.25	0.007
No	39 (25.0)	14 (16.5)	25 (35.2)		
Yes	117 (75.0)	71 (83.5)	46 (64.8)		
Gestational age				χ^2^ = 2.34	0.126
Early preterm (<34 weeks)	58 (37.2)	27 (31.8)	31 (43.7)		
Late preterm (34-36 weeks)	98 (62.8)	58 (68.2)	40 (56.3)		
Number of ANC visits				χ^2^ = 3.13	0.077
<4	78 (50.0)	37 (43.5)	41 (57.7)		
≥4	78 (50.0)	48 (56.5)	30 (42.3)		
Premature rupture of membranes				χ^2^ = 0.01	0.942
No	83 (53.2)	45 (52.9)	38 (53.5)		
Yes	73 (46.8)	40 (47.1)	33 (46.5)		
Number of vaginal examinations				χ^2^ = 0.34	0.544
≤2	115 (73.7)	61 (71.8)	54 (76.1)		
>2	41 (26.3)	24 (28.2)	17 (23.9)		

Prevalence of urogenital bacterial colonization

**Figure 1:**
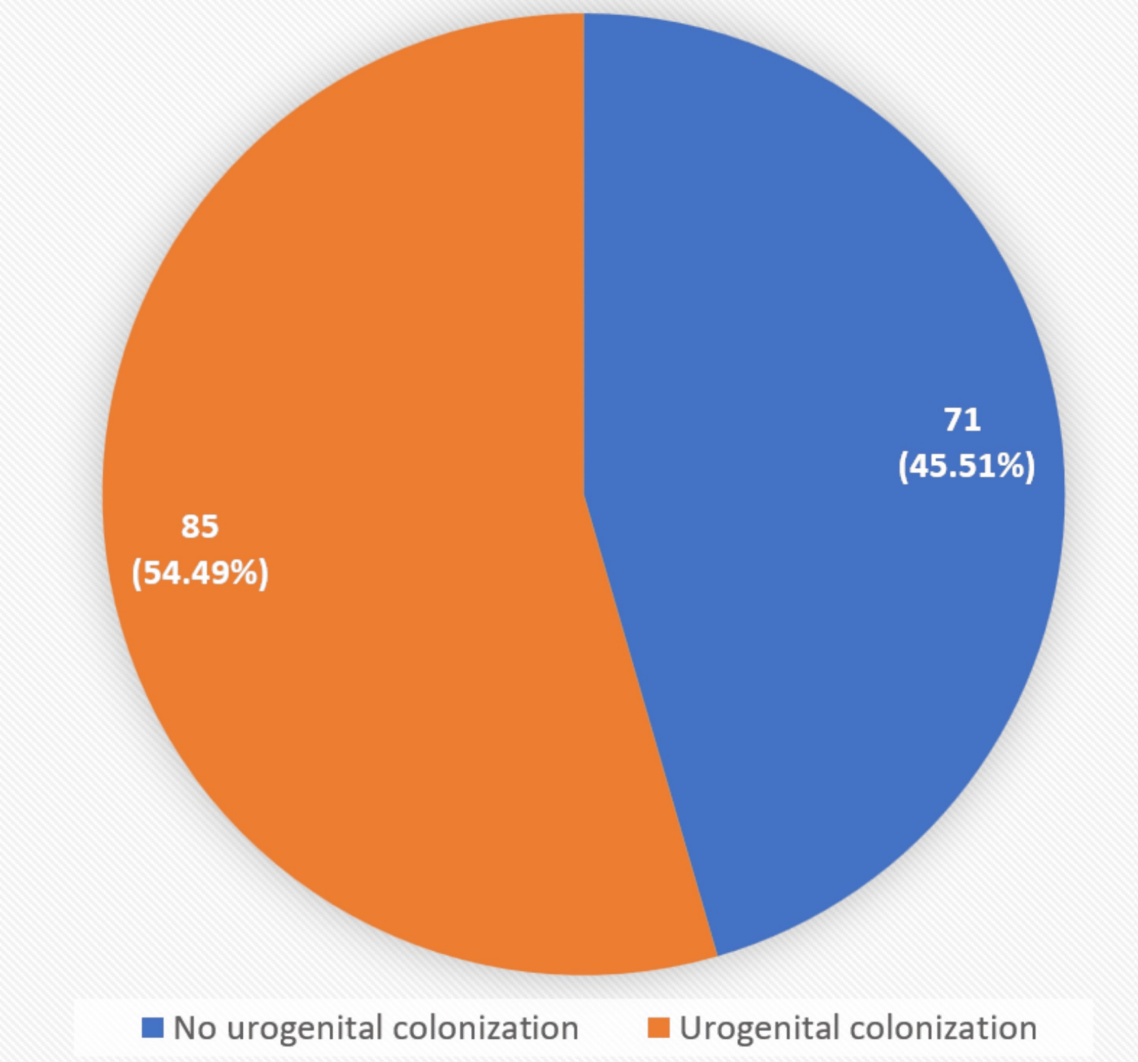
Prevalence of urogenital bacterial colonization among women with preterm labor at MRRH (N = 156) MRRH: Mbarara Regional Referral Hospital

The overall prevalence of bacterial colonization among women with preterm labor at MRRH was 54.5% (95% CI: 46.56-62.19) (Figure 1). Genital tract colonization was more common than urinary tract colonization (48.1% vs. 26.9%, p < 0.001). Overall, 33 women (21.2%) had bacterial colonization of both the urinary and genital tracts.

Frequency of bacterial isolates

Bacteria were isolated from urine samples of 43 participants (27.6%) of the 156. *Klebsiella* spp. were the most frequently isolated urinary pathogens, accounting for 20 (46.5%) of all urine isolates, followed by *Staphylococcus aureus* (16, 37.2%). *Escherichia coli* was isolated from four participants (9.3%). Among genital tract specimens, *Staphylococcus aureus* was the predominant organism, accounting for 57.3% of all genital tract isolates. In contrast, *Proteus* spp. and *Pseudomonas* spp. were the least frequently isolated organisms, each representing 1.3% of genital tract isolates.

Factors associated with urogenital bacterial colonization

On multivariable analysis, age <20 years and the presence of abnormal vaginal discharge were independently associated with urogenital bacterial colonization among women with preterm labor at MRRH. Compared with women aged 20-34 years, those aged <20 years had a 66% higher prevalence of urogenital bacterial colonization (aPR = 1.66, 95% CI: 1.23-2.25; p = 0.001). Similarly, women with abnormal vaginal discharge had a 79% higher prevalence of urogenital bacterial colonization compared with those without abnormal vaginal discharge (aPR = 1.79, 95% CI: 1.12-2.87; p = 0.016) (Table 3). No statistically significant associations were observed for HIV status, multiple sexual partners, referral status, gestational age, number of antenatal care visits, premature rupture of membranes, or number of vaginal examinations.

**Table 3:** Factors associated with urogenital bacterial colonization among women with preterm labor at MRRH ANC: antenatal care, HIV: human immunodeficiency virus, cPR: crude prevalence ratio, aPR: adjusted prevalence ratio, MRRH: Mbarara Regional Referral Hospital

Variable	Bivariate modified Poisson regression analysis	p-value	Multivariable modified Poisson regression analysis	p-value
	cPR (95%CI)		aPR (95%CI)	
Age category (years)				
20-34	Ref		Ref	
<20	1.48 (1.08-2.03)	0.014	1.66 (1.23-2.25)	0.001
≥35	1.04 (0.61-1.78)	0.875	1.32 (0.73-2.38)	0.362
Multiple sexual partners in past 6 months				
No	Ref		Ref	
Yes	0.68 (0.27-1.68)	0.400	0.88 (0.42-1.83)	0.730
Referral status				
No	Ref		Ref	
Yes	1.12 (0.78-1.61)	0.553	1.14 (0.80-1.62)	0.472
HIV status				
Negative	Ref		Ref	
Positive	0.91 (0.51-1.64)	0.757	1.18 (0.65-2.12)	0.586
Abnormal vaginal discharge				
No	Ref		Ref	
Yes	1.69 (1.08-2.64)	0.021	1.79 (1.12-2.87)	0.016
Gestational age				
Early preterm (<34 weeks)	Ref		Ref	
Late preterm (34-36 weeks)	1.27 (0.92-1.75)	0.144	1.17 (0.86-1.59)	0.332
Number of ANC visits				
<4	Ref		Ref	
≥4	1.30 (0.97-1.74)	0.082	1.19 (0.88-1.60)	0.253
Premature rupture of membranes				
No	Ref		Ref	
Yes	1.01 (0.76-1.35)	0.943	0.94 (0.72-1.24)	0.680
Number of vaginal examinations				
≤2	Ref		Ref	
>2	1.10 (0.81-1.51)	0.534	1.07 (0.80-1.44)	0.648

## Discussion

In this study, more than half of the women presenting with preterm labor had urogenital bacterial colonization. This high prevalence highlights the substantial burden of bacterial colonization among women with preterm labor at a tertiary hospital in southwestern Uganda. The finding is consistent with the established association between urogenital bacterial colonization and preterm labor, in which colonizing microorganisms may ascend into the upper genital tract and trigger inflammatory responses that release cytokines and prostaglandins implicated in the initiation of preterm labor [6].

The prevalence observed in our study is comparable to that reported in studies conducted in Andhra Pradesh, India (50%), and Brazil (49%) among women with preterm labor [11,17]. The similarity may be explained by comparable study designs, recruitment of women presenting with preterm labor, and the use of tertiary-level healthcare facilities serving predominantly low-resource populations.

However, the prevalence in our study was higher than that reported in studies conducted in the Republic of Korea (17.9%) and India (36.5%) [8,18]. Differences in study populations and microbiological sampling techniques may partly explain these findings. For example, the Korean study excluded women with premature rupture of membranes. In contrast, nearly half of the women with bacterial colonization in our study reported liquor drainage before the onset of labor. Additionally, the Indian study relied solely on vaginal swabs and urine cultures, whereas our study included endocervical swabs, which may have increased the likelihood of detecting bacterial colonization.

Women aged <20 years were significantly more likely to have urogenital bacterial colonization than those aged 20-34 years. This finding is consistent with studies conducted in Ghana and Kenya, which reported higher rates of genital tract colonization among younger pregnant women [10,19]. The observed association may be explained by biological and social factors. Adolescent mothers are more likely to have cervical ectopy and an immature genital tract, which may increase susceptibility to colonization by pathogenic microorganisms [20-22]. Additionally, younger mothers often experience socioeconomic disadvantages, poor nutritional status, and limited access to reproductive health services, factors that may increase vulnerability to genital tract bacterial colonization [23-25].

Women with preterm labor who reported abnormal vaginal discharge were approximately 1.8 times more likely to have urogenital bacterial colonization than those without abnormal vaginal discharge. This finding suggests that abnormal vaginal discharge may be an important clinical indicator of underlying bacterial colonization among women with preterm labor. Similar associations have been reported in studies conducted in Brazil and Pakistan, where abnormal vaginal discharge was associated with genital tract infections and adverse pregnancy outcomes [26,27].

The observed association is biologically plausible because abnormal vaginal discharge is a common manifestation of disturbances in the vaginal microbiota and reproductive tract infections. Colonization by pathogenic bacteria may alter the normal vaginal flora, resulting in changes in color, consistency, volume, or odor of vaginal secretions [28]. Consequently, women with abnormal vaginal discharge are more likely to harbor potentially pathogenic microorganisms than those without symptoms. From a clinical perspective, this finding suggests that women presenting with preterm labor and abnormal vaginal discharge should be considered for microbiological evaluation, as they may represent a high-risk group for urogenital bacterial colonization. Additionally, abnormal vaginal discharge may therefore be a useful clinical screening marker in resource-limited settings where routine microbiological testing is not readily available.

Strengths

The major strength of this study was the use of microbial cultures from multiple anatomical sites, including urine, high vaginal swabs, and endocervical specimens, thereby increasing the likelihood of detecting urogenital bacterial colonization. Additionally, the study provides valuable local evidence that can inform clinicians in the management of women presenting with preterm labor. The use of standardized microbiological procedures and appropriate statistical analyses further strengthened the reliability of the study findings.

Limitations

This study has some limitations. It was conducted at a single tertiary referral hospital, which may limit the generalizability of the findings to other settings. Additionally, the study relied on conventional microbial culture methods, which may have failed to detect fastidious or unculturable organisms, potentially underestimating the true prevalence of urogenital bacterial colonization. Furthermore, because of the cross-sectional study design, the identified factors should be interpreted as associations rather than causal relationships.

## Conclusions

Urogenital bacterial colonization was common among women presenting with preterm labor at MRRH, with more than half of the participants had urogenital bacterial colonization. Age <20 years and the presence of abnormal vaginal discharge were independently associated with urogenital bacterial colonization. These findings highlight the need for increased clinical suspicion and targeted evaluation of urogenital bacterial colonization among women with preterm labor, particularly adolescents and those with abnormal vaginal discharge.

## Appendices

Appendix 1: data collection tool (questionnaire)

Study Title

Urogenital Bacterial Colonization: Prevalence, Antibiotic Susceptibility and Associated Factors Among Women With Preterm Labor at Mbarara Regional Referral Hospital

Instructions to the interviewer

● Circle the code(s) of the response(s) given by a respondent from the options provided where necessary.

● Other responses should be written in the spaces/boxes provided.

Identification

Participant ID No: __________________________________________________________

Researcher

Name: __________________________________ Data Clerk __________________________

Date: ___________________________________ Date: ________________________________

**Table 4:** Data collection tool English version CRO: ceftriaxone, CIP: ciprofloxacin, AMP: ampicillin, AZM: azithromycin, CN: gentamicin, CAZ: ceftazidime, AZC: ceftazidime/clavulanic acid, S: sensitive, R: resistant, I: intermediate

No.	Questions				Coding categories/responses				
01	How old are you?				Age in completed years _____				
02	How many pregnancies have you carried beyond 7 months?				_____				
03	Where do you reside? (Urban if residing in municipality/city)				Urban area _____ 0 Rural area _____ 1				
04	Have you been referred to MRRH?				No _____ 0 Yes _____ 1				
05	What is the highest level of school you attended?				None _____ 0 Primary _____ 1 Secondary _____ 2 Tertiary/university _____ 3				
06	What is your marital status				Single _____ 0 Married/living with a partner _____ 1 Divorced/separated _____ 2				
07	Have you changed partners in the last 6 months?				No _____ 0 Yes _____ 1				
08	Gestational age (calculate from LNMP or 1^st^ trimester obstetric ultrasound scan)				_____ weeks + _____ days				
09	Number of ANC visits attended (cross check with ANC attendance card/book)				_____				
10	Have you noticed a change in color, volume, or smell of your vaginal discharge?				No _____ 0 Yes _____ 1				
11	Did you get leakage of liquor before labor pains?				No _____ 0 Yes _____ 1				
12	How many times have you been examined vaginally?				_____				
13	HIV status (Either patient knows, or indicated in the file)				HIV negative _____ 0 HIV positive _____ 1				
14	Are you a known patient with diabetes?				No _____ 0 Yes _____ 1				
15	Have you used any antibiotic in the last 7 days?				No _____ 0 Yes _____ 1				
16	Do you have an abnormal vaginal discharge?				No _____ 0 Yes _____ 1				
	Outcome variable								
17	Is there bacterial colonization of the urinary tract? (Obtain from the microbiology report)				No _____ 0 Yes _____ 1				
18	If yes, which organism(s) has been isolated?								
19	Is there bacterial colonization of the genital tract? (Obtain from the microbiology report)				No _____ 0 Yes _____ 1				
20	If yes, which organism(s) has been isolated?								
Culture and sensitivity (record: S or I or R)									
Specimen		Organism	CRO (30 µg)	CIP (5 µg)	AMP (10 µg)	AZM (30 µg)	CN (10 µg)	CAZ (30 µg)	AZC (30/10 µg)
Urine									
HVS									
Endocervical									
